# Genomic Differentiation during Speciation-with-Gene-Flow: Comparing Geographic and Host-Related Variation in Divergent Life History Adaptation in *Rhagoletis pomonella*

**DOI:** 10.3390/genes9050262

**Published:** 2018-05-18

**Authors:** Meredith M. Doellman, Gregory J. Ragland, Glen R. Hood, Peter J. Meyers, Scott P. Egan, Thomas H. Q. Powell, Peter Lazorchak, Mary M. Glover, Cheyenne Tait, Hannes Schuler, Daniel A. Hahn, Stewart H. Berlocher, James J. Smith, Patrik Nosil, Jeffrey L. Feder

**Affiliations:** 1 Department of Biological Sciences, University of Notre Dame, Notre Dame, IN 46556, USA; gregory.ragland@ucdenver.edu (G.J.R.); glenrayhood@rice.edu (G.R.H.); pmeyers2@nd.edu (P.J.M.); scott.p.egan@rice.edu (S.P.E.); powellt@binghamton.edu (T.H.Q.P.); lazorchakp@jhu.edu (P.L.); mglover@nd.edu (M.M.G.); ctait@nd.edu (C.T.); hannes.schuler@boku.ac.at (H.S.); Jeffrey.L.Feder.2@nd.edu (J.L.F.); 2 Environmental Change Initiative, University of Notre Dame, Notre Dame, IN 46556, USA; 3 Department of Integrative Biology, University of Colorado, Denver, CO 80217, USA; 4 Department of Biosciences, Rice University, Houston, TX 77005, USA; 5 Advanced Diagnostics and Therapeutics Initiative, University of Notre Dame, Notre Dame, IN 46556, USA; 6 Department of Biological Sciences, State University of New York, Binghamton, NY 13902, USA; 7 Department of Computer Science, Johns Hopkins University, Baltimore, MD 21218, USA; 8 Department of Entomology and Nematology, University of Florida, Gainesville, FL 32611, USA; dahahn@ufl.edu; 9 Department of Entomology, University of Illinois at Urbana-Champaign, Urbana, IL 61801, USA; stewartb@life.illinois.edu; 10 Lyman Briggs College and Department of Entomology, Michigan State University, East Lansing, MI 48824, USA; jimsmith@msu.edu; 11 Department of Animal and Plant Sciences, University of Sheffield, Sheffield S10 2TN, UK; p.nosil@sheffield.ac.uk

**Keywords:** latitudinal clines, eclosion time, initial diapause depth, apple maggot fly

## Abstract

A major goal of evolutionary biology is to understand how variation within populations gets partitioned into differences between reproductively isolated species. Here, we examine the degree to which diapause life history timing, a critical adaptation promoting population divergence, explains geographic and host-related genetic variation in ancestral hawthorn and recently derived apple-infesting races of *Rhagoletis pomonella*. Our strategy involved combining experiments on two different aspects of diapause (initial diapause intensity and adult eclosion time) with a geographic survey of genomic variation across four sites where apple and hawthorn flies co-occur from north to south in the Midwestern USA. The results demonstrated that the majority of the genome showing significant geographic and host-related variation can be accounted for by initial diapause intensity and eclosion time. Local genomic differences between sympatric apple and hawthorn flies were subsumed within broader geographic clines; allele frequency differences within the races across the Midwest were two to three-fold greater than those between the races in sympatry. As a result, sympatric apple and hawthorn populations displayed more limited genomic clustering compared to geographic populations within the races. The findings suggest that with reduced gene flow and increased selection on diapause equivalent to that seen between geographic sites, the host races may be recognized as different genotypic entities in sympatry, and perhaps species, a hypothesis requiring future genomic analysis of related sibling species to *R. pomonella* to test. Our findings concerning the way selection and geography interplay could be of broad significance for many cases of earlier stages of divergence-with-gene flow, including (1) where only modest increases in geographic isolation and the strength of selection may greatly impact genetic coupling and (2) the dynamics of how spatial and temporal standing variation is extracted by selection to generate differences between new and discrete units of biodiversity.

## 1. Introduction

A major goal of evolutionary biology is to understand how variation within interbreeding demes gets partitioned by natural selection and other evolutionary processes into differences between reproductively isolated species. In this regard, analysis of hybrid zones and resultant clines provides “windows” into adaptive evolution, population divergence, and the speciation process [1,2]. Hybrid zones and clines can originate under different geographic contexts (primary or secondary), can be due to a variety of different factors causing reproductive isolation (RI), such as divergent ecological selection and inherent genetic incompatibilities, and exist along spatial and genomic dimensions [3,4,5,6,7,8,9,10,11,12]. The challenge is therefore to discern from patterns of phenotypic and genetic differentiation, the history, factors, and processes responsible for forming and shaping clines, and how they can act, interact, and become coupled to create and maintain biodiversity [4,7,8,9,11,13,14,15,16,17,18,19,20,21,22,23]. Implications from this endeavor may be strengthened by manipulation, transplant, and crossing experiments testing whether candidate phenotypes and genotypes respond as predicted to selection and cause RI [24,25,26,27]. Thus, hybrid zones and clines provide natural and experimental laboratories to examine how new species arise and are constructed [28]. 

Here, we investigate the genomics of apple and hawthorn-infesting fly populations of *Rhagoletis pomonella* Walsh (Diptera: Tephritidae) by using genotyping-by-sequencing (GBS) data to determine the degree to which the timing of its overwintering pupal diapause explains geographic and host-related differentiation among populations. Our strategy involved coupling a genome wide association study (GWAS) of adult eclosion time [29] and selection experiments on initial diapause intensity [30] with a population survey of four sites arrayed across a 430 km north–south transect where apple and hawthorn flies co-occur (are “sympatric”) in the Midwestern USA (Table S1, Figure S1). Our rationale was that concordance between the genomic responses observed in the GWAS and selection experiments with patterns seen in the population survey provides evidence for the action of selection on diapause timing affecting geographic and host-related clines in the fly. Such concordance allows inferences to be drawn concerning how standing variation may be extracted by local phenological conditions to generate ecologically and genetically differentiated forms in sympatry. 

The recent shift of *R. pomonella* from its ancestral host downy hawthorn (*Crataegus mollis*) to introduced domesticated apple (*Malus domestica*) in the mid-1800s is an example of ecological divergence-with-gene-flow, hypothesized to also represent speciation in action [31,32,33]. Variation in diapause life history timing adapting apple and hawthorn flies to a seasonal difference in the fruiting times of apple vs. hawthorn trees have been shown to be important in generating partial ecologically based RI between the fly “host races” [34,35]. Thus, the apple and hawthorn host races are hypothesized to represent an early stage of differentiation, only partly sorted by divergent selection along the ecological speciation continuum into distinct taxa due to the homogenizing effects of gene flow. In this regard, mark-release-recapture studies have estimated that inter-host migration between sympatric apple- and hawthorn-infesting fly populations occurs at a rate of ~4% per generation [35] and there is no detectable intrinsic RI between the races [36]. Thus, directional selection and drift in geographically and reproductively isolated demes [37,38], rather than divergent selection in the face of gene flow, can be discounted as causes for host-related divergence. The host races therefore can be thought of as constituting a primary hybrid zone of recent historical origin distributed across a spatial mosaic of apple and hawthorn trees interspersed across the landscape. 

Diapause is an important adaptation in many insects to time their life cycle so that individuals are inactive when conditions are unfavorable for growth and reproduction and active when favorable [39,40,41,42,43]. The overwintering pupal diapause in *R. pomonella* is important because the fly is univoltine (has one generation per year) and has a limited adult lifespan of about one month in the field [44]. Thus, flies must time when they terminate diapause and eclose as adults to synchronize their life cycle with the presence of ripe fruit for mating and oviposition (*Rhagoletis* mate only on or near ripe fruit on their host plants [35]). Fruit on apple varieties favorable for *R. pomonella* larval development tend to ripen three–four weeks earlier than those on downy hawthorn [34,35]. As a result, apple flies are shifted in the time that they break diapause to eclose earlier than hawthorn flies to correspond to the earlier fruiting phenology of apples, generating partial allochronic prezygotic mating isolation between the host races at local sites where the flies co-occur [34,35]. 

In addition to diapause termination, host fruiting time also selects on how intensely flies initially enter diapause [45,46]. The pupal diapause is facultative [47]; when exposed to high temperature for an extended period, flies can forgo diapause and directly develop into adults [47]. Such “non-diapause” development is detrimental to fitness, as flies will either eclose when host fruit is not available or start, but not complete, development prior to the onset of cold weather, with many dying during winter. Thus, the earlier fruiting phenology of apple has also been hypothesized to select for a deeper initial diapause depth in the apple than hawthorn fly race [30,45]. 

Host-related divergence in diapause life history timing between local sympatric populations of apple and hawthorn flies is overlaid, however, by broader patterns of geographic variation within the races. The phenologies of apples and hawthorns also vary with latitude, with fruit on both host trees, especially hawthorns, tending to ripen relatively later (after more growing degree days) with decreasing latitude in the Midwest [48]. Consequently, the intensity of initial diapause depth and the timing of adult eclosion also differ geographically within the host races, with populations from further south in the Midwest eclosing later (to adapt to later fruiting times) and having greater initial diapause intensities (to adapt to longer prewinter periods) than flies from northern sites [48,49,50,51]. 

The story of the recent phenological shift of *R. pomonella* to apple is further complicated by evidence suggesting that geographic variation in diapause life history timing in the ancestral hawthorn race has a deep history and may be of secondary origin. Hawthorn-infesting populations of *R. pomonella* appear to have become geographically separated into allopatric isolates in the USA and Mexico ~1.5 million years ago (Mya) [49,52,53,54]. Subsequent episodes of secondary contact and gene flow have been inferred to have generated adaptive latitudinal clines in diapause traits for hawthorn flies across the eastern USA. Since then, the standing diapause variation in hawthorn flies has been hypothesized to facilitate shifts of *R. pomonella* onto a number of different host plants with different fruiting times, including snowberries, blueberries, sparkleberries, flowering dogwood, and most recently apple, generating new sibling species and races in the process [49,52,55]. Thus, not only may diapause life history in *R. pomonella* be differentially selected across geographic and host axes, but there are different historical dimensions of this selection, as well. Specifically, geographic clines in the fly reflect an older, secondary origin, while host-related “genomic” clines are of primary origin associated with the recent shift from hawthorn to apple. 

Previous studies supported several aspects of the diapause cline hypothesis (see Table S2 for synopsis). Doellman et al. [51] showed that the genetic response of 10,241 single nucleotide polymorphisms (SNPs) in the eclosion time GWAS of Ragland et al. [29] explained a significant portion of the geographic variation within the host races across four latitudinally arrayed sites in the Midwest from Grant, MI, to Fennville, MI, to Dowagiac, MI, and Urbana, IL, the sites further analyzed in the current study (Table S1, Figures S1 and S2). Eclosion time was most strongly associated with geographic variation for SNPs mapping to chromosomes 1–3 in the hawthorn race and chromosomes 2 and 3 in the apple race (Table 1) (note: the genome of *R. pomonella* consists of five major chromosomes numbered 1–5 and a small dot chromosome 6 that currently has no mapped loci). Eclosion time could also account for host-related divergence for chromosomes 1–3 between apple and hawthorn flies at the four sympatric sites. 

**Figure 1:**
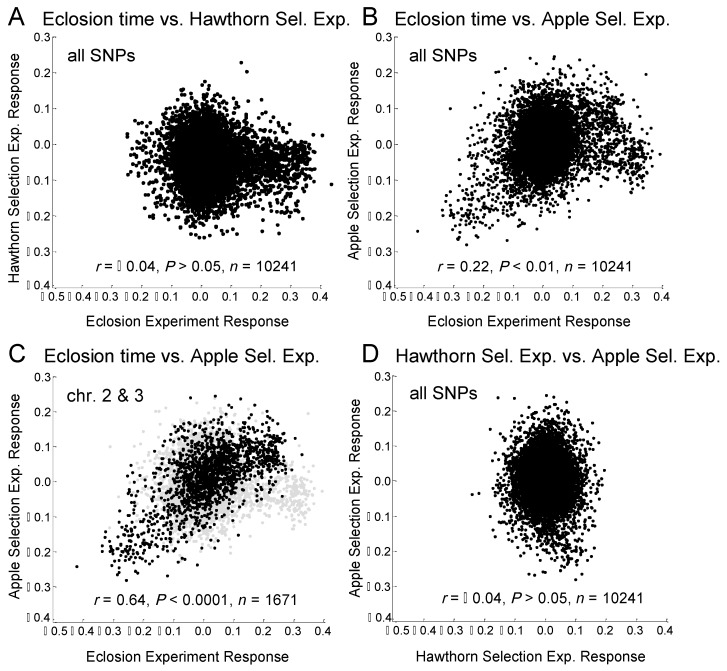
Relationships of the genomic responses of SNPs in the apple prewinter selection experiment (allele frequency difference between treatments, 7–32 days), hawthorn prewinter selection experiment (allele frequency difference between treatments, 7–32 days), and eclosion time GWAS (allele frequency difference between groups, early–late eclosing). (**A**) Eclosion time vs. hawthorn selection experiment for all 10,241 SNPs; (**B**) Eclosion time vs. apple selection experiment for all 10,241 SNPs; (**C**) Eclosion time vs. apple selection experiment for SNPs mapping to chromosomes 2 and 3; and (**D**) Hawthorn vs. apple selection experiment for all 10,241 SNPs. Note that for panel (**C**) the overall relationship for all 10,241 genotyped SNPs is depicted by light grey dots to highlight the subset of SNPs of interest in black for comparison in the graph.

However, Doellman et al. [51] additionally reported that not all geographic variation could be explained by eclosion time (Table 1). It is therefore possible that initial diapause intensity could account for a significant portion of the geographic and host-related divergence not explained by eclosion time. In this regard, Egan et al. [30] conducted a selection experiment on initial diapause intensity for hawthorn flies at the Grant, MI site that involved varying the length of the prewinter period experienced by pupae. They showed that hawthorn flies surviving longer prewinter periods emulating the earlier fruiting time of apples displayed significant genome-wide shifts in SNP frequencies toward the apple race. A corresponding experiment for the apple race still needs to be performed, an objective we accomplish here. Importantly, the genomic response to selection on initial diapause intensity in the hawthorn race was not correlated with that in the eclosion time GWAS of Ragland et al. [29] (*r* = −0.044, *p* = 0.41, *n* = 10,241 SNPs; Figure 1A), implying genetic independence between these “front” and “back” end components, respectively, of diapause timing. The lack of a genetic correlation with eclosion time makes the hypothesis that initial diapause depth could account for unexplained geographic and host-related genetic variation more attractive. 

Our three initial objectives in the current study were to determine: (1) if selection on initial diapause intensity in the apple race follows the same pattern as in the hawthorn race and is largely modular and genomically independent from eclosion time; (2) if regions of the genome showing geographic variation in apple and hawthorn flies that could not be accounted for by eclosion time could be explained by selection on initial diapause intensity; (3) if the extent of local host divergence at sympatric sites, where apple and hawthorn flies co-occur, could be explained by the combination of selection acting on eclosion time and initial diapause depth. Collectively, these three objectives extend analysis of eclosion time [51] to permit consideration of selection acting genome wide on all currently known aspects of diapause affected by host fruiting phenology. 

The new results, when combined with those of Egan et al. [30], Ragland et al. [29], and Doellman et al. [51], allow us to investigate a fourth and overarching objective: to compare the magnitude of primary hybrid zone divergence (i.e., local SNP frequency differences between sympatric populations of the races) to that of secondary geographic differentiation (i.e., SNP frequency differences within the hawthorn race across the range surveyed). This fourth objective thus provides insight into the extent to which divergent natural selection has transformed standing latitudinal variation in diapause timing into distinguishable genotypic differences between the host races in sympatry. In this regard, speciation may be viewed as the process by which differentiated sets of genes evolve causing RI between populations. Eventually populations diverge to pass a threshold or “tipping point” to become new species, at which time they may be distinguishable as different genetic clusters of individuals that retain their distinction if, when, or where they happen to co-occur [56,57,58]. Under this perspective, a key question is understanding how RI transitions from primarily being a consequence of the direct action of natural selection on individual genes (“genic effects”) to a more collective property of all divergently selected genes in the genome [59]. As this happens, the effects of selection on genes become coupled across loci as linkage disequilibrium (LD) builds up between populations to create multi-locus barriers to gene flow [4,7,8,13,14,15,16,17,18,20,21,23,60,61]. As a result, the genome begins to congeal, as whole genotypes of migrants tend to be eliminated from populations before individual genes can introgress [58,62,63,64]. The current study lets us examine how far the *R. pomonella* host races have progressed in this process, both locally at sympatric sites and globally across the geographic landscape.

## 2. Materials and Methods

### 2.1. Geographic Survey

Data on geographic variation are those from Doellman et al. [51]. Adult flies genotyped in the survey were reared in the laboratory from larval-infested apple and downy hawthorn fruit collected at four sympatric field sites across the Midwestern USA where host trees and the fly races co-occurred within 1 km of each other (Table S1, Figure S1).

### 2.2. Eclosion Study

Details on the eclosion time GWAS performed on apple and hawthorn flies from the Fennville, MI site can be found in Ragland et al. [29]. Genome wide, SNP frequency responses in eclosion time were highly correlated between apple and hawthorn flies (*r* = 0.54, *p* < 0.0001 for all 10,241 SNPs genotyped; *r* = 0.66, *p* < 0.0001 for 4244 of these SNPs mapped to chromosomes 1–5). We therefore present results for the mean eclosion time responses of SNPs averaged between the host races in the analyses performed in the current study.

### 2.3. Prewinter Selection Experiments

Egan et al. [30] investigated the effect that initial diapause intensity has on genome wide differentiation in the hawthorn race by GBS. Here, we extend the prewinter selection experiment to apple flies using the same study design implemented for hawthorn flies. Larval-infested apple fruit collected at the Grant, MI site in 1989 were transported to the lab and flies reared to pupation and placed under long (32 days) versus short (seven days) prewinter conditions at 25 °C. After 30 weeks of cold (4 °C) to simulate winter, the pupae were returned to 21 °C to emulate post-winter (spring) warming. Eclosing flies that survived were collected as adults and genotyped by GBS (*n* = 41 for the 32-day treatment, *n* = 48 for the seven-day treatment, roughly equally divided by sex). Thus, the long prewinter treatment applied selection on initial diapause intensity, presumably selecting against flies that underwent non-diapause development, whereas the more benign seven-day treatment provided a control sample.

### 2.4. Genotyping-by-Sequencing

Methods for GBS of short DNA sequence reads of individually barcoded double digest restriction-site associated DNA libraries (ddRAD-seq), de novo genome assembly of contigs, variant SNP calling, and allele frequency estimation were done following Egan et al. [30] and Ragland et al. [29]. Sequencing was performed on HiSeq platforms, generating >1.2 billion 100 to 124-bp reads. We used custom scripts and the Genome Analysis Toolkit (GATK version 2.5-2; [65]) to identify a common set of 10,241 variable SNP sites passing quality filters that were genotyped in the eclosion study, in the apple and hawthorn prewinter selection experiments, and at all four paired sympatric sites. Average SNP coverage per individual was 6.2× in the eclosion time GWAS, 4.8× in the hawthorn prewinter selection experiment, 4.6× in the apple prewinter selection experiment, and 3.3× in the geographic survey of sympatric sites.

### 2.5. Linkage Disequilibrium and Inversion Polymorphism

*Rhagoletis* has a highly-structured genome that complicates interpretation of the GBS results. In particular, previous studies have provided evidence for inversion polymorphism on all five major chromosomes of the *R. pomonella* genome [29,30,66,67]. Details concerning the intricacies of the structure of the inferred chromosomal rearrangements in the fly are presented in Egan et al. [30], Ragland et al. [29], and Doellman et al. [51]. To take LD and genome structure into account, in addition to testing all 10,241 genotyped SNPs and all 4244 mapped variants, we also separately analyzed three different classes of linked SNPs on each chromosome categorized in Ragland et al. [29] as displaying high, intermediate, or low levels of composite LD [68] with one another. Briefly, the program LDna (linkage disequilibrium network analysis) of Kemppainen et al. [69] was used to generate groups of SNPs displaying an *r*^2^ value of >0.6 with at least one other member of the group (high LD class), SNPs with a *r*^2^ value <0.15 with any other SNPs residing on the same chromosome (low LD class), and SNPs possessing *r*^2^ values ranging from 0.15 to 0.6 with linked loci (intermediate LD class). Presumably, these classes represent varying degrees of association with inversion polymorphisms on each chromosome; SNPs in the high LD class are located within putative inverted regions, while low LD class SNPs are in collinear regions. Intermediate LD SNPs are more weakly associated with the inversions, lying either just outside of the inverted region or within the inversion, but experiencing occasional gene flux. 

### 2.6. Tests for Genetic Response in Eclosion Time, Prewinter Selection Experiments, Geographic Variation, and Host-Related Differentiation

Probabilities of single locus genotypes and allele frequencies for SNPs were calculated following McKenna et al. [70]. Tests for significant allele frequency differences for SNPs were performed using a non-parametric, Monte Carlo approach between sample populations of: (1) early vs. late eclosing flies; (2) seven-day vs. 32-day prewinter treatments in the apple and hawthorn selection experiments; (3) apple and hawthorn populations from Grant, MI vs. Urbana, IL, the difference representing the extent of latitudinal geographic variation within the host races across the Midwestern USA; and (4) local pairs of sympatric apple and hawthorn fly populations. Two randomized samples of whole fly genotypes were drawn with replacement from the pool of the two sample populations being tested, corresponding to the respective numbers of flies genotyped in each sample. Allele frequency differences were then calculated between the random samples and the process repeated 10,000 times to generate expected null distributions, with the use of whole fly genotypes preserving LD relationships among loci in the analyses. An observed absolute difference for a SNP that exceeded the absolute upper 95th quantile of the null Monte Carlo distribution was taken as statistically significant. To determine if overall table-wise percentages of SNPs displaying significant associations with eclosion time, initial diapause depth, and geographic variation were greater than expected by chance, the total percentage of SNPs showing significant differences in each simulation was determined and the distribution for all 10,000 runs for a given test compared to the actual percentages to assess if they were in the upper 95th quantile of simulated values, following Ragland et al. [29].

### 2.7. Tests for Genetic Associations of Eclosion Time, Prewinter Selection Experiments, Geographic Variation, and Host-Related Differentiation

We performed linear regression analyses in R [71] to assess the degree to which SNP frequency differences in the eclosion time GWAS, and apple and hawthorn selection experiments explained geographic variation within the host races between the Grant and Urbana sites and host-related divergence between the races at the four sympatric sites. In addition, we also performed analyses determining how latitudinal geographic variation between Grant and Urbana within the apple and hawthorn races was related to host-associated differentiation between apple and hawthorn flies at the four sympatric sites. Significance levels were determined by Monte Carlo simulations in which regressions were calculated between random samples of individual whole fly genotype probabilities taken with replacement from the appropriate pooled data sets of flies. Observed *r*^2^ values in regressions that exceeded the upper 5% of values in the simulated distribution in 10,000 replicates were taken as significant. 

To quantify the extent to which diapause life history timing explains patterns of geographic and host-related genomic differentiation in *R. pomonella*, we performed forward and backward multiple regressions including four explanatory variables: SNP frequency responses in the eclosion time GWAS and the apple and hawthorn selection experiments, along with mean LD values for SNPs to all other linked loci. Separate analyses were conducted for the following response variables: (1) frequency differences between hawthorn or apple populations from Grant and Urbana; and (2) frequency differences between hawthorn and apple flies at each of the four sympatric sites. Regressions were performed using the ‘stepAIC’ function in the R package ‘MASS’ [72], using the 4244 SNPs that have been mapped in the *R. pomonella* genome for which mean LD values could be calculated to take into account the effects of genetic associations between SNPs.

### 2.8. Tests for Genetic Clustering among Populations

Tests for evidence of genetic subdivision were performed by Discriminant Analysis of Principal Components (DAPC) using the R package ‘adegenet‘ v2.0.0 [73,74]. DAPC is well-suited for *Rhagoletis* because it is not affected by loci displaying high levels of genetic association (LD) with one another, as compared to methods like STRUCTURE [75], as it uses orthogonal principal components (PCs) of the genetic data to assess the degree to which individuals are correctly assigned (membership) into pre-defined populations (designated k). We ran DAPC, considering all 10,241 SNPs genotyped in the study, separately for each pair of apple and hawthorn fly populations at the four sympatric sites (k = 2), to test for local host-related clustering. We also assessed hawthorn flies from the Grant and Urbana sites (k = 2), to test for geographic clustering within the hawthorn race between the ends of the range surveyed in the Midwest. Finally, to explicitly compare levels of geographic versus host-related clustering, we ran DAPC for hawthorn and apple flies at the Grant and Urbana sites (k = 4). Results are presented for the smallest number of PCs found to show the best discrimination between designated populations based on their a-scores, the difference between the proportion of successful reassignment of individuals to correct populations in the analysis (observed discrimination) and values obtained using randomized groups (random discrimination) adjusted for the number of PCs analyzed. We tested for significant genetic clustering of populations by Monte Carlo simulation. Individuals were randomly assigned to predesignated populations and DAPC performed based on the same number of PCs used in the original analysis. Observed values of mean probabilities for correct assignment of individuals to populations were then compared to simulated values (*n* = 10,000 reps) to determine if they exceeded the upper 5% of the randomized distributions.

We also examined the question of local (sympatric) and global (geographic) genetic clustering for the host races in two additional ways. First, we constructed unrooted neighbor-joining networks from overall Nei’s genetic distances [76] calculated between populations using the ‘poppr’ v.2.4.1 and ‘ape’ v4.1 packages in R [77,78,79]. Bootstrap support values for nodes in the networks were calculated based on 10,000 replicates across loci. Networks were constructed based on genetic distance estimates calculated for all 10,241 SNPs genotyped in the study, as well as for the 3276 SNPs displaying significant responses in the eclosion time GWAS and/or prewinter selection experiments (i.e., the subset of loci most likely affected by selection on diapause life history timing). Second, we tested for isolation-by-distance (IBD) and isolation-by-ecology (IBE) among populations based on Nei’s genetic distance estimates between apple and hawthorn fly populations at the same and different sites by Mantel tests [71]. Geographic distance was considered in terms of the difference in the latitudinal order of populations from one another along the transect in the Midwest and ecological distance as whether the populations being compared infested the same (0) or different hosts (1). Given our sampling scheme of collecting paired apple and hawthorn fly populations at four sympatric sites, the variable of geography and ecology did not co-vary between analyses. Nei’s genetic distances between populations were randomly permuted 10,000 times keeping geographic and host variables constant to determine whether observed genetic correlations with geographic distance and host were expected less than 5% by chance.

### 2.9. Analysis of Linkage Disequilibrium among Unlinked Loci

We calculated composite LD values for unlinked loci according to Weir [68] to examine the extent to which selection acts on the genome as a unit to differentiate apple and hawthorn flies. To assess this, we calculated LD between SNPs mapping to different chromosomes: (1) within local apple and hawthorn fly populations at the four sympatric sites; (2) between the host races based on pooled apple and hawthorn fly populations at the four sympatric sites; and (3) between hawthorn fly populations at Grant and Urbana. We restricted our analysis to SNPs (*n* = 524) displaying significant responses in the eclosion time GWAS and/or prewinter selection experiments, and that showed a genetic response (allele frequency difference) of at least 0.2 in one of the three studies (i.e., loci likely to be under strong diapause-related selection). These comparisons allowed us to examine whether and the extent to which selection has acted in a concerted manner across the genome between host plants and across geography to elevate LD among unlinked SNPs above baseline levels within the races, a key metric of progress towards coupling/speciation.

## 3. Results

### 3.1. Genetic Response in the Apple Prewinter Selection Experiment

**Figure 2:**
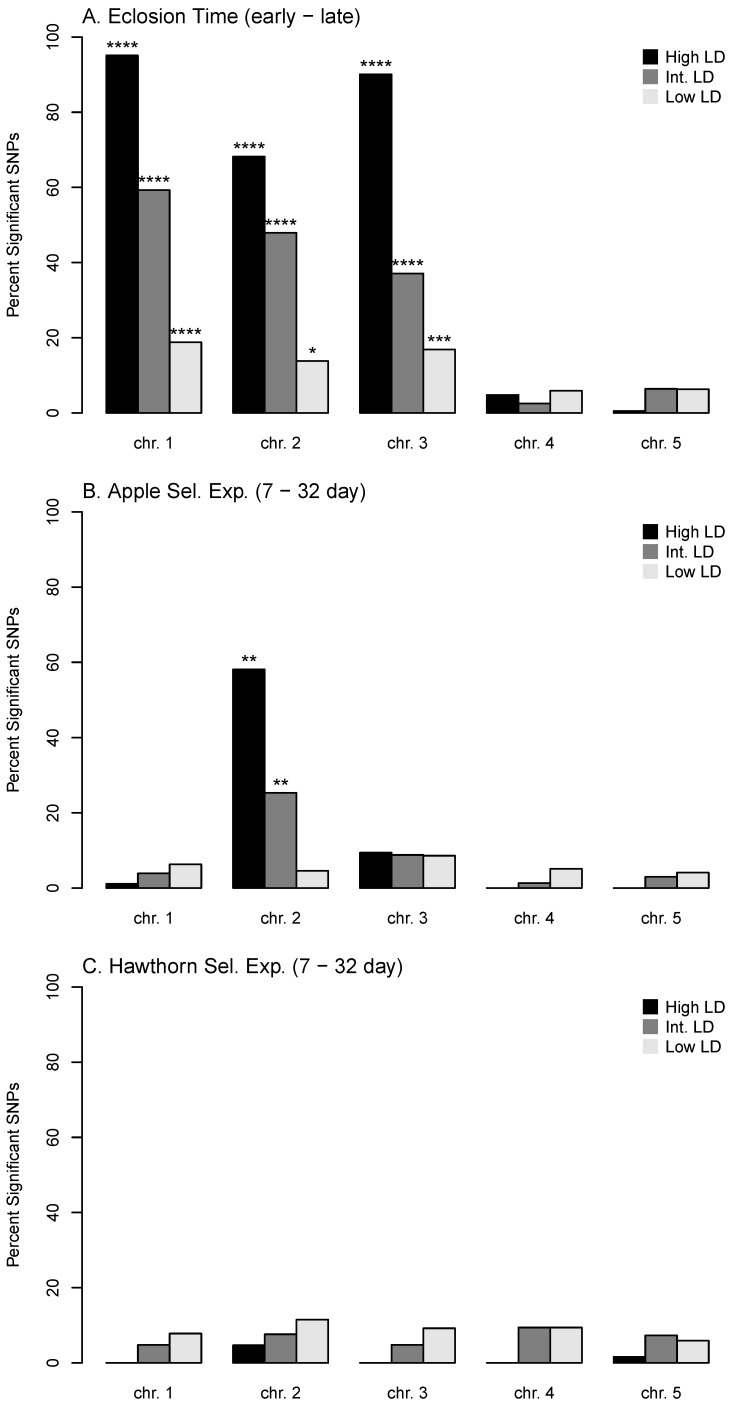
Percentages of SNPs displaying significant responses in each diapause experiment. (**A**) Eclosion time GWAS; (**B**) Apple prewinter selection experiment; and (**C**) Hawthorn prewinter selection experiment. Results are given for each chromosome separately, for High, Intermediate (Int.), and Low LD SNP classes. * = *p* < 0.01; ** = *p* < 0.01; *** = *p* < 0.001; **** = *p* < 0.0001.

The genomic response in the apple prewinter selection experiment was concentrated on chromosome 2, and, in particular, in the high and intermediate LD classes of loci on chromosome 2 (Figure 2B; Table S3). There was also a relatively high percentage, although not a significant excess, of responding SNPs on chromosome 3 (8.9% = 89/996). No other class of SNPs on chromosomes 1, 4, or 5, however, contained a percentage of significant loci in the apple prewinter experiment greater than 6.3%.

### 3.2. Association of Genetic Responses among Apple and Hawthorn Prewinter, and Eclosion Experiments

In contrast to the hawthorn selection experiment [29], SNP frequency differences between the seven-day and 32-day prewinter treatments in the apple fly selection experiment were significantly positively related to the differences between the earliest and latest quantiles of eclosing flies in the GWAS (*r* = 0.22, *p* < 10^−2^, *n* = 10,241; Figure 1B). The significant correlation was driven by chromosome 2 (*r* = 0.68, *p* < 10^−4^, *n* = 675; Table S4) and chromosome 3 (*r* = 0.51, *p* < 10^−2^, *n* = 996; Table S4, see Figure 1C for graph of the combined results for chromosomes 2 and 3). The remainder of SNPs on chromosomes 1, 4, and 5 did not show a significant relationship between the apple selection experiment and eclosion time (*r* = −0.03, *p* = 0.48, *n* = 2573; Table S4). The lack of a relationship for chromosome 1 was of note because this linkage group showed a strong response in the eclosion time GWAS but not in the apple selection experiment (Figure 2A,B; Table S3). 

Overall, no relationship existed between the apple and hawthorn selection experiments (*r* = −0.04, *p* = 0.41, *n* = 10,241; Figure 1D). However, as was true for eclosion time [29], a significant negative relationship existed between the apple and hawthorn selection experiments for the high LD SNPs on chromosome 2 (*r* = −0.39, *p* < 0.05, *n* = 129; Table S4).

### 3.3. Relationship of Apple Prewinter Selection Experiment with Geographic Variation

**Figure 3:**
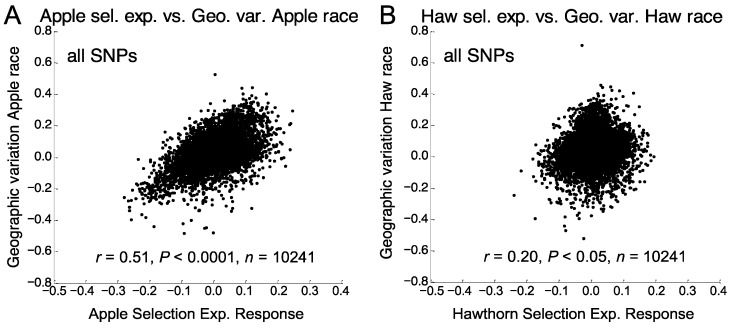
Relationships of the genomic responses of SNPs in the prewinter selection experiments (allele frequency difference between treatments, 7–32 days) vs. geographic variation between the Grant, MI and Urbana, IL sites (allele frequency difference between sites, Grant—Urbana) within the host races for all 10,241 SNPs genotyped in the study. (**A**) Apple selection experiment vs. geographic variation within the apple race; (**B**) hawthorn selection experiment vs. geographic variation within the hawthorn race.

The apple selection experiment was significantly related to the pattern of geographic variation genome-wide between the Grant and Urbana sites within both the apple (*r* = 0.51, *p* < 10^−4^, *n* = 10,241, Figure 3A) and hawthorn race (*r* = 0.23, *p* < 0.05, *n* = 10,241). As was the case for eclosion time (Table S5; [51]), the genomic responses for chromosomes 2 and 3 in the apple selection experiment were strongly associated with geographic variation in the apple (*r* for chromosomes 2 and 3 combined = 0.72, *p* < 0.0001, *n* = 1671) and hawthorn race (*r* = 0.65, *p* < 0.0001, *n* = 1671; Table S6). However, unlike the results for eclosion time (Table S5; [51]), the apple selection experiment also significantly explained geographic variation for chromosome 1 in the apple race (*r* = 0.30, *p* < 0.05, *n* = 949; Table S6). In addition, the apple selection experiment accounted for geographic variation in the apple race for low LD SNPs mapping to chromosomes 1, 3, and 4 (*r* combined = 0.40, *p* < 0.0001, *n* = 537), while the eclosion time GWAS did not (*r* combined = 0.07, *p* > 0.05, *n* = 537; Table S5; [51]). The same was also true for the high LD class of loci in the apple race mapping to chromosomes 4 and 5 (*r* combined = 0.40, *p* < 0.0001, *n* = 416; compare Tables S5 and S6). The high LD loci on chromosome 4 are notable because these SNPs collectively showed elevated geographic variation in the apple, but not the hawthorn race (Table 1).

### 3.4. Relationship of Hawthorn Prewinter Selection Experiments with Geographic Variation

The genetic response in the hawthorn selection experiment was significantly related to geographic variation genome-wide in the hawthorn race (*r* = 0.20, *p* = 0.029, *n* = 10,241; Figure 3B), but not the apple race (*r* = −0.01, *p* > 0.05, *n* = 10,241). As was true for eclosion time and the apple selection experiment, the genetic response in the hawthorn selection experiment was positively related to geographic variation displayed by chromosome 3 in the hawthorn race (Table S7). In addition, the hawthorn selection experiment was significantly related to geographic variation displayed by the low LD class of loci on chromosomes 3, 4, and 5 in the hawthorn race (*r* combined = 0.39, *p* < 0.0001, *n* = 630; Table S7). These SNPs are notable because they represent the only loci that showed increased geographic variation in the hawthorn race that could not be related to eclosion time or the apple selection experiment (Table 1, Tables S5 and S6). 

### 3.5. Relationship of Eclosion Time and Prewinter Selection Experiments with Geographic Variation

Multiple regressions of the apple and hawthorn selection experiments and eclosion time, in concert with LD, explained 54.0% and 38.2% of genome-wide genetic variation within the hawthorn and apple races, respectively, between Grant and Urbana (Table 2). All four predictors comprised the best model for the hawthorn race, with each showing a significant positive relationship with geographic variation between Grant and Urbana. For the apple race, eclosion time, the apple selection experiment, and LD significantly and positively predicted geographic variation between Grant and Urbana and were retained in the best model. 

### 3.6. Diapause, Geographic, and Host Race Differentiation at Sympatric Sites

**Figure 4:**
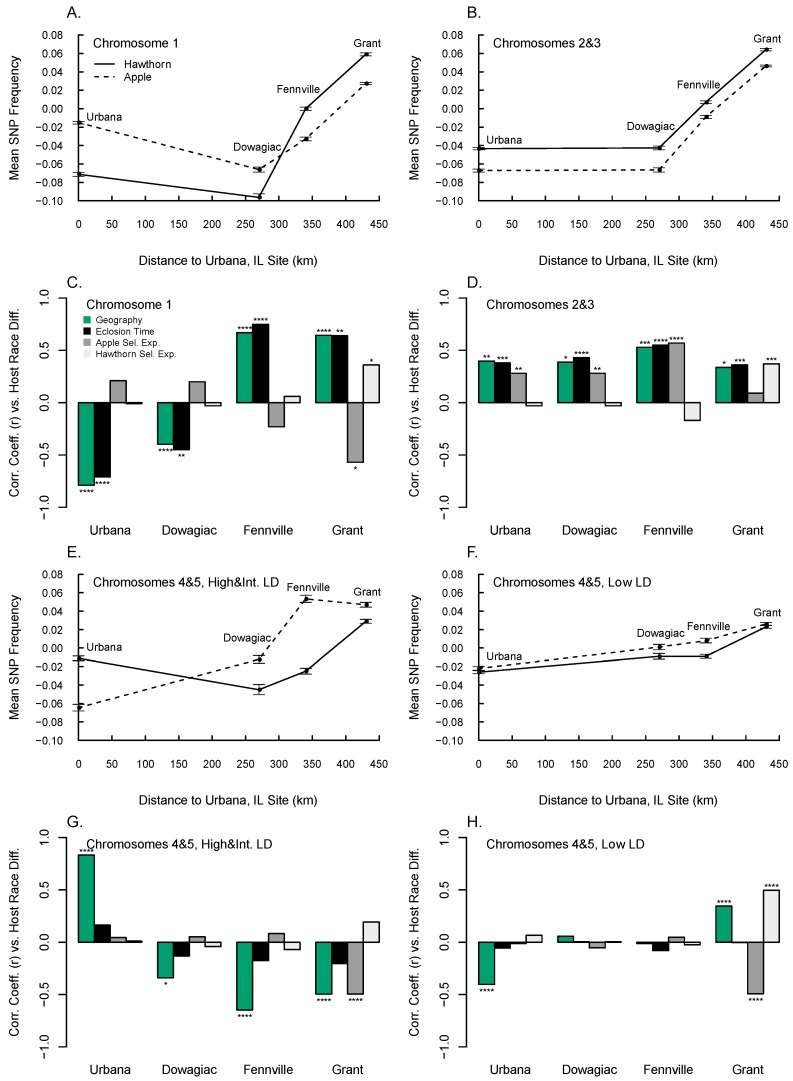
Mean SNP allele frequencies (±SE) for apple (dashed line) and hawthorn (solid line) fly races across the four sympatric study sites (panels **A**,**B**,**E**,**F**) and correlation coefficients (r) between host-related allele frequency divergence at the four sympatric sites versus geographic variation (Grant–Urbana; green bars) and the genetic response in the eclosion time (black bars), apple prewinter (dark grey bars), and hawthorn prewinter (light grey bars) selection experiments (panels **C**,**D**,**G**,**H**). Graphs are shown for: (**A**,**C**) Chromosome 1; (**B**,**D**) Chromosomes 2 and 3 combined; (**E**,**G**) Chromosomes 4 and 5 high and intermediate LD SNPs combined; and (**F**,**H**) Chromosomes 4 and 5, low LD SNPs combined. Mean SNP frequency for populations represent the average difference for all SNPs in the category being considered from the mean of the races between the Grant and Urbana sites (zero value), using the more common allele at Grant as a positive reference. Sample sizes for correlations involving chromosome 1, chromosomes 2 and 3, chromosomes 4 and 5 (high and intermediate), and chromosomes 4 and 5 (low) are 949, 1671, 201, and 456, respectively. * = *p* < 0.05; ** = *p* < 0.01; *** = *p* < 0.001; **** = *p* < 0.0001.

The eclosion time and selection experiments, as well as geographic variation, were significantly related to SNP frequency differences between hawthorn and apple flies at the four sympatric sites (Figure 4). However, the relationship was dependent upon the chromosome, site, and experiment being considered. We therefore present the results below assessing chromosome 1 (Figure 4A,C), chromosomes 2 and 3 (Figure 4B,D), the high and intermediate LD SNPs on chromosomes 4 and 5 (Figure 4E,G) and the low LD SNPs on chromosomes 4 and 5 (Figure 4F,H) separately, that epitomize how the different studies variably affected host-associated divergence across the four sympatric sites. 

Chromosome 1 displayed greater geographic variation in hawthorn than apple flies, generating a crossing pattern of SNP frequencies for the host races between Grant and Urbana (Figure 4A). As a result, the pattern of host-related divergence for chromosome 1 SNPs differed across sites, with variants present in higher frequency in the hawthorn than apple race at Grant and Fennville tending to be found at lower frequency at Dowagiac and Urbana. Thus, the sign of the relationship between host divergence and both eclosion time and geographic variation within the hawthorn race for chromosome 1 was significantly positively associated at Grant and Fennville, while being negatively associated at Dowagiac and Urbana (Figure 4C). The apple selection experiment displayed the reverse pattern in explaining host-associated divergence for chromosome 1 than eclosion time, being negatively related at Grant and Fennville and positive at Dowagiac and Urbana. However, the apple selection experiment was not as strong a predictor of host divergence for chromosome 1 as eclosion time, with only the relationship at Grant being statistically significant. The hawthorn selection experiment significantly explained host-associated divergence for chromosome 1 only at the Grant site (positive relationship). 

For chromosomes 2 and 3, allele frequencies collectively varied in a parallel clinal manner for hawthorn and apple flies, resulting in a consistent pattern of local host-related differentiation across all four sympatric sites (Figure 4B). Both geographic variation (averaged between host races) and eclosion time were significant positive predictors of SNP frequency differences between the host races at sympatric sites (Figure 4D). As the genetic response in the apple selection experiment for chromosomes 2 and 3 was positively correlated with eclosion time (*r* = 0.64, *p* < 10^−4^, *n* = 1671; Figure 1C), the apple selection experiment also significantly predicted host divergence at sites, with the only exception being Grant. In comparison, the response in the hawthorn selection experiment for chromosomes 2 and 3 was not highly associated with that in the eclosion time GWAS (*r* = −0.17, *p* > 0.05, *n* = 1671) or apple selection experiment (*r* = −0.12, *p* > 0.05, *n* = 1671). Thus, as with chromosome 1, the hawthorn selection experiment significantly explained host-related divergence only at the Grant site. 

High and intermediate LD SNPs on chromosomes 4 and 5 displayed a crossing pattern of SNP frequencies for the host races between Grant and Urbana, with a steeper cline in the apple race (Figure 4E). Geographic variation within the apple race between Grant and Urbana was significantly associated with host race differentiation at all four sites, with a strong positive relationship at Urbana and a negative relationship at the remaining sites (Figure 4G). Host race differentiation within the high and intermediate LD SNPs on chromosomes 4 and 5 showed no association with diapause traits, with the exception of a negative relationship with the apple selection experiment at Grant. 

The low LD SNPs on chromosomes 4 and 5 generally showed the least geographic variation, host-associated divergence, and differences in eclosion time (Figure 4F,H; Table 1 and Table S3). As a result, eclosion time did not significantly account for host differences for low LD SNPs on chromosomes 4 and 5 at any of the four pairs of apple and hawthorn fly sites (Figure 4H). However, the hawthorn and apple selection experiments did significantly explain host divergence for low LD SNPs on chromosomes 4 and 5 at Grant, being significantly positively related for the hawthorn selection experiment and negatively related for the apple selection experiment. In addition, geographic variation within the hawthorn host race was negatively correlated with host differences at Urbana, but positively correlated with host differences at Grant. 

Multiple regressions of eclosion time and the hawthorn and apple selection experiments, in concert with LD, significantly explained genome-wide variation between apple and hawthorn flies at all four sympatric sites (Table 3). At Grant and Fennville, all four factors were significant in the best models, which explained 34.8% and 28.8% of sympatric host race differentiation, respectively. The best model explained 4.3% of host race differentiation at Dowagiac and included eclosion time and the apple selection experiment, while 9.7% of host race differentiation at Urbana was explained by eclosion time, the apple selection experiment, and LD.

### 3.7. Local and Global Patterns of Genetic Differentiation

The magnitude of local host divergence between apple and hawthorn flies at sympatric sites was subsumed within the broader geographic patterns displayed within the races. Overall Nei’s genetic distances between Grant and Urbana apple and hawthorn fly populations based on all 10,241 SNPs scored in the study were 0.0124 and 0.0138, respectively. These values were about twice as large as the mean distances between the races at the four sympatric sites (0.0065 ± 0.013 s.e., *n* = 4). The results were similar when considering the 3276 SNPs showing significant differences in the eclosion time GWAS or prewinter selection experiments, a subset of loci likely affected by selection on diapause life history (Nei’s genetic distance between Grant and Urbana apple fly populations = 0.020; hawthorn fly populations = 0.027; mean distance between the races at the four sympatric sites = 0.0090 ± 0.013 s.e., *n* = 4). 

Isolation-by-distance existed among fly populations across the Midwest. The difference in latitudinal order of collecting sites was significantly related to Nei’s genetic distances between populations for all 10,241 SNPs scored in the study (*r* = 0.55, *p* < 0.003, 27 df, Mantel test) and the 3276 SNPs showing significant responses in the eclosion time GWAS or prewinter selection experiments (*r* = 0.54, *p* < 0.002). However, there was no evidence for isolation-by-ecology, as host affiliation was not significantly related to Nei’s genetic distances between populations for all 10,241 SNPs (*r* = −0.15, *p* = 0.452) or for the 3276 SNPs displaying significant responses in the eclosion time GWAS or prewinter selection experiments (*r* = −0.14, *p* = 0.282).

### 3.8. Genetic Clustering among Populations

**Figure 5:**
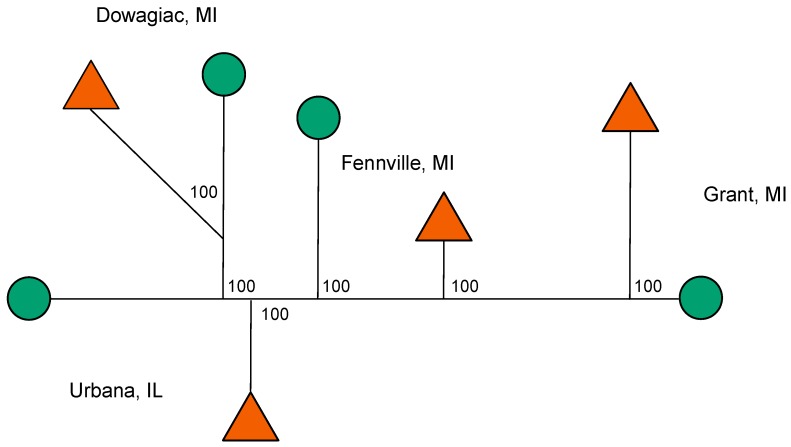
Unrooted neighbor-joining network for apple (green circles) and hawthorn (red triangles)-infesting fly populations from Grant, MI, Fennville, MI, Dowagiac, MI, and Urbana, IL based on Nei’s genetic distances calculated for all 10,241 SNPs genotyped in the study. Bootstrap support values, calculated from 10,000 replicates across loci, were 100 for all nodes in the network.

The host races showed no evidence of clustering globally across the Midwest (Figure 5). Indeed, the neighbor joining network for all 10,241 SNPs tended to genetically group local apple and hawthorn populations at sympatric sites together, with high bootstrap support of 100%, rather than the host races across sites (Figure 5). The network was essentially identical for the 3276 SNPs displaying significant responses in the eclosion time GWAS or prewinter selection experiments, except that branch lengths were longer (data not shown).

**Figure 6:**
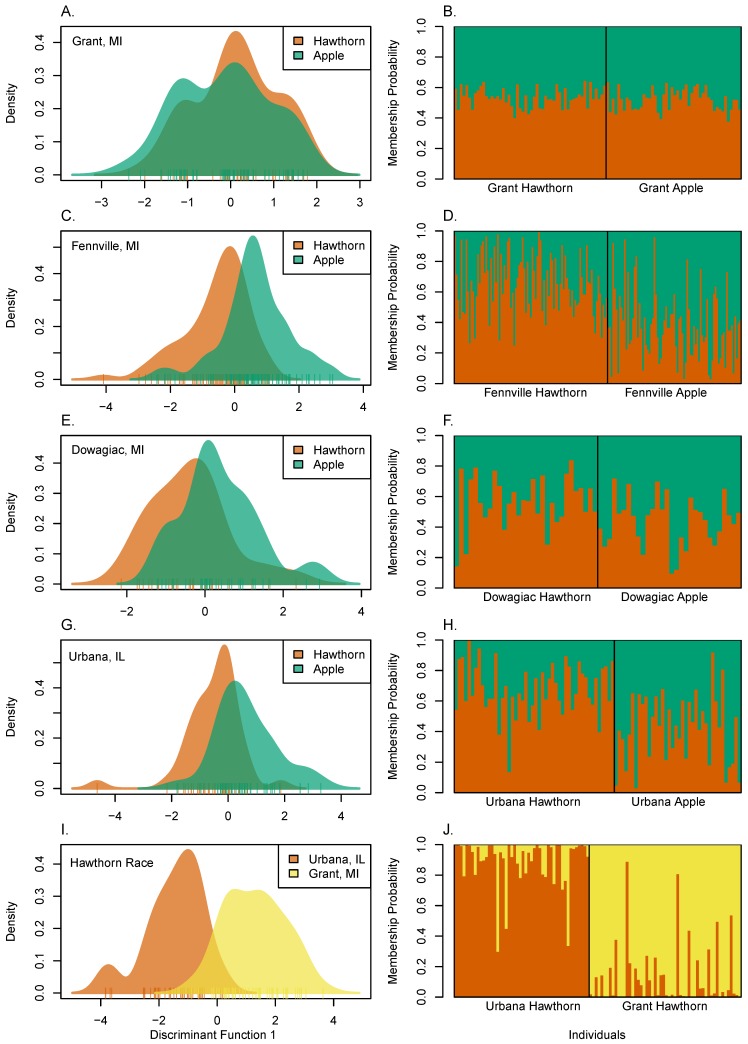
Discriminant Analysis of Principal Components (DAPC) discriminant function plots (panels **A**,**C**,**E**,**G**,**I**) and assignment probabilities (panels **B**,**D**,**F**,**H**,**J**) for individual flies analyzed for k = 2 predesignated populations of: (**A**,**B**) apple and hawthorn flies from Grant, MI (number of Principal Components [PC] as determined by a-scores = 1; mean assignment probability to correct population = 0.510 ± 0.007 s.e.; *p* = 0.183 for significant genetic clustering, as determined by Monte Carlo simulation); (**C**,**D**) apple and hawthorn flies from Fennville, MI (number of PCs = 5; mean assignment probability = 0.642 ± 0.016 s.e.; *p* ≤ 0.001); (**E**,**F**) apple and hawthorn flies from Dowagiac, MI (number of PCs = 4; mean assignment probability = 0.565 ± 0.021 s.e.; *p* = 0.089); (**G**,**H**) apple and hawthorn flies from Urbana, IL (number of PCs = 8; mean assignment probability = 0.633 ± 0.022 s.e.; *p* = 0.001); and (**I**,**J**) hawthorn flies from Grant, MI and hawthorn flies from Urbana, IL (number of PCs = 14; mean assignment probability = 0.886 ± 0.018 s.e.; *p* ≤ 0.001).

With respect to the sympatric sites, the DAPC analysis revealed that apple and hawthorn flies show a range of local host-related genomic clustering (Figure 6A–H). At the lower end of divergence at Grant (Figure 6A,B), limited and statistically non-significant clustering was detected between apple and hawthorn flies (mean probability of correct assignment of an individual to a race = 0.510 ± 0.007 s.e.; *p* = 0.182, as determined by Monte Carlo simulations). At the upper end of local divergence at Fennville (Figure 6C,D), modest and statistically significant clustering was observed (mean probability of correct membership assignment to a race = 0.642 ± 0.016; *p* < 0.0001). However, even at Fennville, the distributions of the host races broadly overlapped and many apple and hawthorn flies were still assigned to their non-natal race at the site (22.4%). In comparison, the hawthorn fly populations from Grant and Urbana (Figure 6I,J) showed stronger levels of geographic clustering than was observed locally between sympatric populations of the host races (mean probability of correct membership assignment to a race = 0.886 ± 0.018 s.e.; *p* < 0.0001). As a result, a much lower proportion of hawthorn flies were assigned to the wrong site between Grant and Urbana (5.9%) than apple and hawthorn flies to the wrong race at Fennville; *p* < 0.001; Fisher’s exact test).

**Figure 7:**
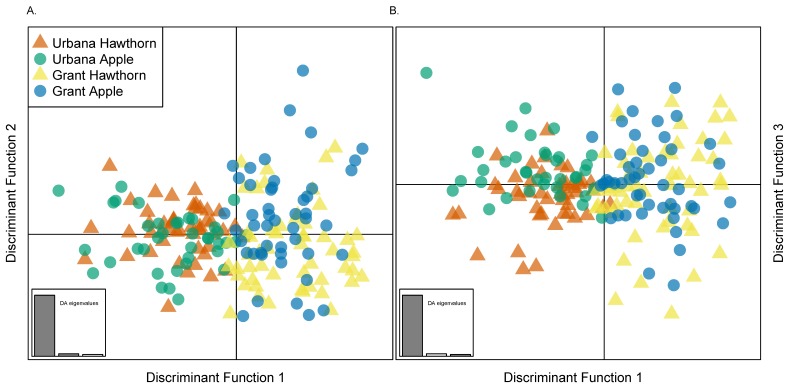
DAPC plot for: (**A**) discriminant functions 1 vs. 2; and (**B**) discriminant functions 1 vs. 3, for individual apple and hawthorn flies from Grant, MI and Urbana, IL. Note: the first discriminant function clusters flies by geography, while second and third discriminant functions show modest effects in differentiating flies at the Grant and Urbana sites, respectively, by host affiliation. Inset plots show a histogram of discriminant analysis (DA) eigenvalues, providing a relative measure of the ratio of between vs. within group variation explained by discriminant functions 1–3 for the PCs retained in the DAPC analysis, which are 93.4%, 3.85%, and 2.75%, respectively.

Discriminant analysis (DAPC) of apple and hawthorn populations from Grant and Urbana (Figure 7) qualified the relative scale of local host-related versus geographic variation and the lack of global geographic clustering for the races. Discriminant function 1, which accounted for 93.4% of the genetic variation explained by the PCs retained in the analysis, principally separated the Grant and Urbana sites geographically from one another (Figure 6A,B). In comparison, discriminant function 2 (3.85% of the genetic variation) and discriminant function 3 (2.75% of the genetic variation) very modestly distinguished the host races from each other locally at the Grant (Figure 7A) and Urbana (Figure 7B) sites, respectively.

### 3.9. Analysis of Linkage Disequilibrium among Unlinked Loci

**Figure 8:**
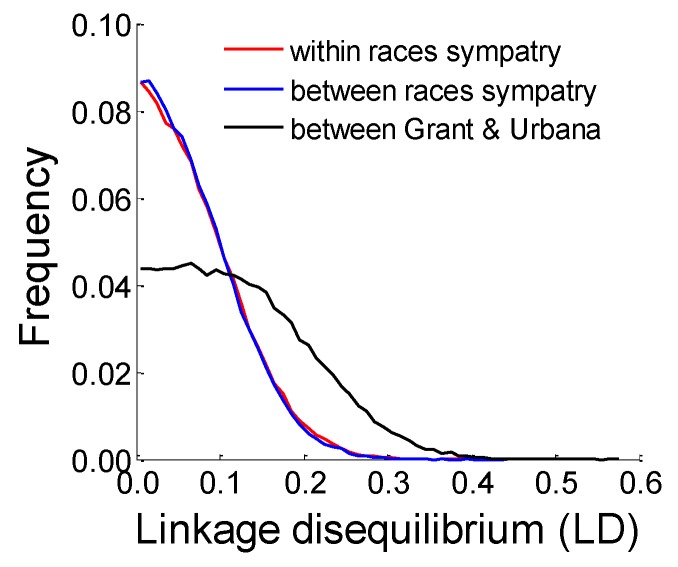
Distributions of composite LD values calculated between unlinked SNPs mapping to different chromosomes that displayed a strong association with diapause (i.e., showed a significant allele frequency difference of >0.2 in the eclosion time GWAS, or the apple or hawthorn selection experiments). A total of 524 SNPs conformed to this criterion (chromosome 1 = 273 SNPs, chromosome 2 = 132 SNPs, to chromosome 3 = 117 SNPs, and chromosome 5 = 2 SNPs. The red curve represents the distribution of mean LD values calculated within the apple and hawthorn fly populations at the four sympatric sites. The blue curve represents the distribution of mean of LD values calculated between the apple and hawthorn fly populations at the four sympatric sites (i.e., estimates of LD derived from pooling host races at sites). The black curve represents distribution of mean of LD values between hawthorn fly populations at the Grant, MI and Urbana, IL sites (i.e., estimates of LD derived from pooling hawthorn fly populations across the two sites).

Analysis of levels of LD between the host races further implied that the genomes of apple and hawthorn flies are not distinct units. Levels of LD between unlinked SNPs most strongly significantly associated with diapause timing in the eclosion time GWAS and/or prewinter selection experiments were virtually the same between versus within the races host at sympatric sites (compare blue and red curves, respectively, in Figure 8). In contrast, a shift toward higher LD was observed between hawthorn populations from Grant vs. Urbana (black curve in Figure 8).

## 4. Discussion

The current study had three initial objectives. With regard to objective 1, our results show that the relationship between initial diapause intensity and eclosion time in the apple race differs from that in the hawthorn race (Figure 1). Our findings indicate that genetic co-variation exists between initial diapause intensity and eclosion time in the apple race, principally for chromosomes 2 and 3. In contrast, Ragland et al. [29] reported that in the hawthorn race eclosion time and initial diapause intensity were largely genetically independent.

With respect to objective 2, regions of the genome showing geographic variation in apple and hawthorn flies that could not be accounted for by eclosion time could be largely explained by the apple and hawthorn selection experiments (Table 1). Indeed, all but one region of the genome showing significant geographic variation above null expectation can be accounted for by the combination of the genetic responses in the eclosion time, and apple and hawthorn selection experiments (Tables S5–S7). The only exception is the intermediate LD class of SNPs on chromosome 4. Thus, it appears that diapause life history timing can largely account for spatial (clinal) variation in *R. pomonella*. Indeed, multiple regressions of the eclosion time, and apple and hawthorn selection experiments, in concert with LD, explained 54.0% and 38.2% of genome-wide genetic variation within the hawthorn and apple races, respectively, between Grant and Urbana (Table 2). Moreover, the direction of allele frequency change in the eclosion time GWAS and prewinter selection experiments concurred with the pattern of geographic variation displayed by the host races. Specifically, in all cases where the genomic response for a chromosome or LD class in the eclosion time, apple or hawthorn selection experiment was significantly related to geographic variation in the apple or hawthorn race, the sign of the relationship was positive (Tables S5–S7). In other words, the SNP in higher frequency in later eclosing flies or in the longer 32-day prewinter treatment tended to be the SNP in higher frequency in both of the host races at the Urbana compared to Grant site, as predicted by the tendency of host trees to require more growing degree days for fruit to ripen at southern sites.

In regard to objective 3, the eclosion time study and apple and hawthorn selection experiments could account for local divergence between the races (Figure 4). Multiple regressions of eclosion time, and the selection experiments, in concert with LD, explained 34.8%, 28.8%, 4.3% and 9.7% of genome-wide variation between apple and hawthorn flies at the Grant, Fennville, Dowagiac, and Urbana, respectively (Table 3).

Taken together, our results allow for an assessment of overarching objective 4, equating primary hybrid zone divergence with secondary geographic differentiation. Our rationale was that such a comparison would provide insight into the extent to which divergent natural selection has transformed standing latitudinal variation in diapause timing into distinguishable, host-related genetic clusters in sympatry. Our conclusions were strengthened in this regard by two aspects of the experimental design and results. First, we combined GWAS and selection experiments on the genomics of phenotypes shown by previous ecological studies to be under divergent selection [34,45], with surveys of natural fly populations. Thus, we could make a strong case that specific SNP and gene regions were affected by selection, rather than just drawing inferences based on the outlier status of loci. Second, a large proportion of geographic and host-related genomic variation could be accounted for by diapause life history timing. Thus, our equating of the genomics of phenological adaptation from the level of local sympatric race differences to the broader geographic scale was valid.

We found that, for SNPs associated with diapause timing, mean levels of geographic variation within the host races across the Midwest are two to three-fold greater than those between apple and hawthorn flies at local sympatric sites. The Grant and Urbana sites are physically separated by 430 km. Thus, assuming that the distribution of host plants is similar at sites resulting in equivalent rates of interhost migration, the mean phenological difference between local apple and hawthorn trees equate to a difference of roughly 193.5 km geographically in the apple race and 143.3 km in the hawthorn race. The degree of genetic divergence between hawthorn populations from Grant vs. Urbana is on a scale at which flies from these two sites show clear evidence for genetic clustering (Figure 6I,J and Figure 7). In comparison, sympatric populations of apple and hawthorn flies show a range of divergence in which clustering is just emerging as an inchoate property of divergent selection on diapause (Figure 6A–H). Thus, depending upon the location, the host races appear to be below or nearing the cusp of levels of population divergence and LD where selection could start coupling the effects of unlinked loci to appreciably increase the barrier strength to gene flow. However, if local differences were enhanced two to three-fold to levels seen between Grant and Urbana, then coupling could potentially act to select against migrant flies at the whole genome level and generate more readily distinguishable genetic clusters of apple and hawthorn flies in sympatry. Future studies of related sibling species in the *R. pomonella* group attacking flowering dogwood, blueberries, sparkleberries, and snowberries that fruit at different times of the season will provide further insight into how selection on diapause translates polymorphism within populations into divergence between taxa and further clarify where the apple and hawthorn host races reside along the speciation continuum.

Although our findings addressed the major objectives of the study, they also raise several questions. One issue concerns reconciling the GWAS eclosion study with the pattern of local host divergence for chromosomes 2 and 3. For these two linkage groups, SNPs associated with later eclosion time were found at higher frequency within both host races at more southern sites, as predicted. However, at sympatric sites, apple flies generally possessed higher frequencies of chromosome 2 and 3 SNPs associated with later eclosion time (Figure 4D), this despite apple flies eclosing earlier, not later, than hawthorn flies in both nature and under controlled laboratory conditions [35,50]. Moreover, although most pronounced for high LD SNPs, this pattern was similar for all LD classes. Consequently, even if low LD SNPs genotyped in the study showed the “expected” pattern, their effects would not be masked by alleles in the inversion going in the “wrong” direction. Thus, the anomaly for eclosion time between the races could not be due only to the unexpected behavior of loci in inversions on chromosomes 2 and 3 dominating the analysis.

Part of the explanation for the apparent anomaly concerning eclosion time may involve the genetic correlation that exists between adult eclosion time and initial diapause intensity for chromosomes 2 and 3 in the apple race. Our results indicate that these two aspects of diapause are positively genetically related for these two chromosomes, such that apple flies that enter deeper initial pupal diapauses have higher frequencies of SNPs that subsequently are associated with later adult eclosion time (Table S4). Thus, it is possible that strong selection on initial diapause intensity in apple flies due to pleiotropy and/or linkage results in their having higher frequencies of SNPs for late eclosion. If this hypothesis is true, then it would suggest that other loci or unresolved epistatic or gene-by-environment interactions are responsible for the earlier eclosion time of apple than hawthorn flies at sympatric sites.

In this regard, chromosome 1 represents the other linkage group in the genome displaying a strong relationship with eclosion time (Figure 2A; Table S3). Like chromosomes 2 and 3, SNPs mapping to chromosome 1 showed pronounced geographic variation within the hawthorn race related to eclosion time, with alleles for later eclosion more common in southern hawthorn flies, as predicted (Table S5; [51]). Unlike chromosomes 2 and 3, however, geographic variation was less prominent within the apple race for chromosome 1 (Figure 4A). In addition, initial diapause depth was not genetically associated with eclosion time for chromosome 1 in the apple race (Table S4), suggesting that these two aspects of diapause may be uncoupled and free to evolve independently with respect to chromosome 1. Consequently, the apple selection experiment significantly accounted for geographic variation in the apple race, with alleles correlated with deeper initial diapause intensity found in higher frequency in more southern apple fly populations, as expected (Table S6). However, geographic variation for chromosome 1 SNPs associated with eclosion time was generally flat and not significant in the apple race (Table 1), resulting in a crossing pattern between the host races (Figure 4A). Consequently, apple flies tended to possess higher frequencies of earlier eclosion alleles than hawthorn flies at the Dowagiac and Urbana sites (Figure 4C), possibly explaining why apple flies at these two sites eclose sooner than hawthorn flies. However, this was not the case at Fennville and Grant, and, thus, it must still be determined why apple flies at the two more northern sites eclose earlier in the season.

A second unresolved issue concerns why eclosion time and initial diapause intensity appear to genetically covary strongly in the apple but not hawthorn race (Figure 1; Table S4). One possible explanation for the difference is that the relationship is not functional but reflects a difference in linkage. In the apple race, loci associated with greater initial diapause intensity for chromosomes 2 and 3 may be in LD with those responsible for later eclosion time, while in the hawthorn race they are not. However, for SNPs displaying significant genetic responses in the eclosion time study and/or apple selection experiment, pairwise composite LD values were strongly positively correlated between the host races at the Grant site for both chromosome 2 (*r* = 0.79, *p* < 10^−10^, *n* = 366 SNPs) and chromosome 3 (*r* = 0.91, *p* < 10^−10^, *n* = 498 SNPs). Thus, genes affecting eclosion time and initial diapause intensity appear to be in the same linkage phase between the host races, arguing against the variable LD hypothesis.

Given the similar linkage phase of SNPs, genes affecting initial diapause intensity and eclosion time may act similarly between the host races, but for some reason were differentially selected in the apple versus hawthorn prewinter experiment. For example, it is possible that harsher environmental conditions experienced by apple than hawthorn-infesting larvae in the field prior to collection and transport to the lab may have resulted in stronger selection for initial diapause intensity in the apple than hawthorn prewinter experiment to the point that loci having pleiotropic effects on eclosion were also selected in the former but not latter study. Until further details on the physiology, pathways, and genes determining initial diapause depth and their relationship to diapause termination and adult eclosion are known, we can only speculate on how ecological differences, as well as possible epistatic and gene-by-environment interactions, may have differentially affected apple versus hawthorn flies in the selection experiments.

Finally, we have assumed that the apple and hawthorn prewinter experiments actually selected on initial diapause intensity. It is possible that a portion of the responding SNPs in these studies were selected for a correlated trait instead (e.g., increased desiccation tolerance). If true, then this could account for why the apple and hawthorn selection experiments differed. For example, it may be that relative humidity levels were lower prior to or during the hawthorn than apple selection experiment and the difference actually selected more strongly for desiccation tolerance than initial diapause intensity in hawthorn flies. Further study is therefore required to determine whether and how variation in relative humidity interacts (or does not) with diapause to genetically affect the host races. In this regard, Ragland et al. [80] has shown that respiration rates vary among individual pupae as they enter diapause, providing a means in future studies to associate responding SNPs in the selection experiments with initial diapause intensity and investigate and distinguish the possible genetic effects of desiccation.

In conclusion, we have been able to associate a significant portion of geographic and host-related divergence in *R. pomonella* to variation in host fruiting phenology and its effects on different aspects of diapause life history timing or on a correlated phenotype affected by prolonged prewinter heating. Selection on diapause timing results in divergent ecological adaptation and a hybrid cline of primary origin between the host races, generating allochronic reproductive isolation RI between local apple and hawthorn fly populations, with the allelic composition of the adaptation changing (sliding) latitudinally across sites. Thus, the recently derived apple-infesting race of *R. pomonella* represents a new taxon partially extracted from broader geographic clines of standing variation present in the ancestral hawthorn race that appear to have formed originally by secondary contact [49,52]. The identities of the specific loci and pathways affecting diapause timing in *Rhagoletis*, however, must still be resolved. In addition, it remains to be determined how selection on diapause life history transforms the partial and variable patterns of divergence observed between the apple and hawthorn host races into locally and geographically discrete and more fully reproductively isolated sibling species in the *R. pomonella* group. Finally, it must be seen how general the results for *Rhagoletis*, in which past and recent history and genome structure contribute to standing variation enabling rapid adaptive diversification, are for other model systems of ecological speciation (see reviews in [12,61,81,82,83,84]). However, our findings concerning the way selection and geography interplay, how host adaptation equates to geography, and that geography is not everything because it subsumes other patterns, could be general for many cases of earlier stages of divergence-with-gene flow. In particular, the implication that only modest increases in geographic isolation and the strength of divergent selection may greatly impact genetic coupling and the genomic clustering of populations could be of broad significance for understanding the dynamics of how spatial and temporal standing variation is extracted by selection to generate differences between new and discrete units of biodiversity.

## Figures and Tables

**Table 1:** Percentages of single nucleotide polymorphisms (SNPs) displaying significant geographic allele frequency differences between apple race (upper table) and hawthorn (lower table) fly populations from Grant, MI vs. Urbana, IL. Results are given for All Mapped SNPs (Map SNP), and for High, Intermediate (Int.) and Low linkage disequilibrium (LD) classes of SNPs for each chromosome separately, as well as all together (chr 1–5). Shaded boxes represent classes of loci having percentages of geographically varying SNPs significantly above null random expectation, as determined by Monte Carlo simulations. Letters designate classes of loci displaying significant regressions between SNP frequency differences in the eclosion time genome wide association study (GWAS) (E), and apple (A) and hawthorn (H) prewinter selection experiments versus geographic variation (see Tables S5–S7 for regression values). Dark grey boxes are classes of loci with significant excesses of geographically varying SNPs that show significant regressions with the genetic response in the eclosion time study, while classes in light grey boxes show significant geographic variation that cannot be explained by eclosion time.

**Apple Race**	**chr 1**	**chr 2**	**chr 3**	**chr 4**	**chr 5**	**chr 1–5**
Map SNP	10 A	39 EA	43 EA	29 A	9	24 EA
High LD	7 EA	57 EA	86 EA	79 A	7 A	33 EA
Int. LD	11 A	39 EA	35 EA	40	11	24 EA
Low LD	10 A	15 EA	15 A	13 A	8	11 A
**Haw Race**	**chr 1**	**chr 2**	**chr 3**	**chr 4**	**chr 5**	**chr 1–5**
Map SNP	54 E	50 EA	48 EA	12	15	37 EAH
High LD	92 E	82 EA	84 EH	5	17	58 EA
Int. LD	46 E	47 EA	42 EAH	12	14	35 EA
Low LD	16 E	21 EA	21 H	15 H	16 H	17 EH

**Table 2:** Multiple regressions for geography. Frequency differences between hawthorn or apple populations from Grant and Urbana were predicted by SNP frequency responses in the eclosion time GWAS and the apple and hawthorn selection experiments, along with mean LD values for SNPs to all other linked loci (LD). The adjusted *R*^2^, *F* statistic and *p*-value for the best model are included, along with the coefficients, standard errors (SE), *t* statistics, and associated *p*-values for each retained component.

**Hawthorn Race**						
**Predictor**	***R*^2^**	**Coeff.**	**SE**	***t***	***F*_4,4239_**	***p***
Eclosion Exp.		0.629	0.013	49.5		<0.0001
Hawthorn Sel. Exp.		0.413	0.023	17.7		<0.0001
Apple Sel. Exp.		0.258	0.016	16.6		<0.0001
LD		0.036	0.005	6.8		<0.0001
Total	0.540				1248	<0.0001
**Apple Race**						
**Predictor**	***R*^2^**	**Coeff.**	**SE**	***t***	***F*_3,4240_**	***p***
Eclosion Exp.		0.185	0.014	13.4		<0.0001
Apple Sel. Exp.		0.698	0.017	40.9		<0.0001
LD		0.046	0.006	8.2		<0.0001
Total	0.3824				876.8	<0.0001

**Table 3:** Multiple regressions for host race. Frequency differences between hawthorn and apple populations from Grant, Fennville, Dowagiac, and Urbana were predicted by SNP frequency responses in the eclosion time GWAS and the apple and hawthorn selection experiments, along with mean LD values for SNPs to all other linked loci (LD). The adjusted *R*^2^, *F* statistic and *p*-value for the best model are included, along with the coefficients, standard errors (SE), *t* statistics, and associated *p*-values for each retained component.

**Grant, MI**						
**Predictor**	***R*^2^**	**Coeff.**	**SE**	***t***	***F*_4,4239_**	***p***
Eclosion Exp.		0.236	0.008	31.3		<0.0001
Hawthorn Sel. Exp.		0.394	0.014	28.5		<0.0001
Apple Sel. Exp.		−0.154	0.009	−16.7		<0.0001
LD		0.008	0.003	2.5		0.012
Total	0.348				565.8	<0.0001
**Fennville, MI**						
**Predictor**	***R*^2^**	**Coeff.**	**SE**	***t***	***F*_4,4239_**	***p***
Eclosion Exp.		0.371	0.012	31.6		<0.0001
Hawthorn Sel. Exp.		−0.061	0.022	−2.8		0.0048
Apple Sel. Exp.		0.050	0.014	3.5		0.0005
LD		0.021	0.005	4.3		<0.0001
Total	0.288				429.4	<0.0001
**Dowagiac, MI**						
**Predictor**	***R*^2^**	**Coeff.**	**SE**	***t***	***F*_2,4241_**	***p***
Eclosion Exp.		−0.034	0.012	−2.8		0.0051
Apple Sel. Exp.		0.239	0.017	13.9		<0.0001
Total	0.043				96.1	<0.0001
**Urbana, IL**						
**Predictor**	***R*^2^**	**Coeff.**	**SE**	***t***	***F*_3,4240_**	***p***
Eclosion Exp.		−0.217	0.013	−16.1		<0.0001
Apple Sel. Exp.		0.281	0.017	17.0		<0.0001
LD		0.020	0.005	3.6		<0.0001
Total	0.097				153.5	0.0004

## Supplementary Materials

The following are available online at http://www.mdpi.com/2073-4425/9/5/262/s1, Figure S1: Map of the ranges and paired sympatric collecting sites for apple and hawthorn host races of *Rhagoletis pomonella*; Figure S2: Relationships between eclosion time and geographic variation within the apple and hawthorn host races; Table S1: Sampling information for paired sympatric collecting sites for apple and hawthorn host races of *Rhagoletis pomonella*; Table S2: Synopsis of prior work and sources of additional data sets analyzed in the current study; Table S3: Percentages of SNPs displaying significant responses in the eclosion time and prewinter selection experiments; Table S4: Correlation coefficients of allele frequency responses in the eclosion time and prewinter selection experiments with each other; Table S5: Correlation coefficients of allele frequency responses in the eclosion time experiment with geographic variation within the apple and hawthorn host races; Table S6: Correlation coefficients of allele frequency responses in the apple prewinter experiment with geographic variation within the apple and hawthorn host races; Table S7: Correlation coefficients of allele frequency responses in the hawthorn prewinter experiment with geographic variation within the apple and hawthorn host races.

## Data Accessibility

All new sequence and SNP data generated from this study are deposited in DRYAD (https://doi.org/10.5061/dryad.q9t27n9).
