# Supplementary material for: Genomic Differentiation during Speciation-with-Gene-Flow: Comparing Geographic and Host-Related Variation in Divergent Life History Adaptation in Rhagoletis pomonella
Source: Genes (Basel). 2018 May 18;9(5):262. doi: 10.3390/genes9050262 (PMC5977202; doi:10.3390/genes9050262)
Supplement: Supplementary file 1 [file genes-09-00262-s001.zip › DiapauseSelectionTableS5.docx]

**Table S5.** Correlation coefficients (r) of SNP frequency differences between early and late eclosing flies in the eclosion time GWAS versus geographic differences between apple race (upper table) and hawthorn race (lower table) populations from Grant, MI and Urbana, IL, as determined in Doellman et al. [51], for All Mapped SNPs (Map SNP), and for High, Intermediate (Int.), and Low LD classes of SNPs. Results are given for each chromosome considered separately, as well as all together (chr 1-5). ^*^ = P < 0.05; ^**^ = P < 0.01; ^***^ = P < 0.001; significant positive relationships are shaded in grey. n = # of SNPs genotyped in the class.

| **Apple race** | **chr 1** | **chr 2** | **chr 3** | **chr 4** | **chr 5** | **chr 1-5** |
| --- | --- | --- | --- | --- | --- | --- |
| Map SNP | n = 949 | n = 675 | n = 996 | n = 436 | n = 1188 | n = 4244 |
|  | 0.07 | 0.72^****^ | 0.72^****^ | 0.14 | 0.02 | 0.42^***^ |
| High LD | n = 263 | n = 129 | n = 223 | n = 42 | n = 374 | n = 1031 |
|  | 0.49^****^ | 0.85^****^ | 0.37^***^ | 0.02 | 0.06 | 0.48^***^ |
| Int. LD | n = 558 | n = 459 | n = 599 | n = 159 | n = 593 | n = 2368 |
|  | 0.05 | 0.65^****^ | 0.63^****^ | 0.13 | -0.01 | 0.39^****^ |
| Low LD | n = 128 | n = 87 | n = 174 | n = 235 | n = 221 | n = 845 |
|  | -0.05 | 0.43^**^ | 0.15 | 0.03 | -0.03 | 0.09 |
| **Haw race** | **chr 1** | **chr 2** | **chr 3** | **chr 4** | **chr 5** | **chr 1-5** |
| Map SNP | n = 949 | n = 675 | n = 996 | n = 436 | n = 1188 | n = 4244 |
|  | 0.82^****^ | 0.73^****^ | 0.69^****^ | 0.04 | 0.11 | 0.72^****^ |
| High LD | n = 263 | n = 129 | n = 223 | n = 42 | n = 374 | n = 1031 |
|  | 0.59^****^ | 0.86^****^ | 0.41^****^ | 0.17 | -0.03 | 0.84^****^ |
| Int. LD | n = 558 | n = 459 | n = 599 | n = 159 | n = 593 | n = 2368 |
|  | 0.75^****^ | 0.67^****^ | 0.61^****^ | -0.09 | 0.10 | 0.63^****^ |
| Low LD | n = 128 | n = 87 | n = 174 | n = 235 | n = 221 | n = 845 |
|  | 0.37^***^ | 0.44^**^ | 0.19 | 0.10 | 0.01 | 0.18^***^ |
