# Supplementary material for: Genomic Differentiation during Speciation-with-Gene-Flow: Comparing Geographic and Host-Related Variation in Divergent Life History Adaptation in Rhagoletis pomonella
Source: Genes (Basel). 2018 May 18;9(5):262. doi: 10.3390/genes9050262 (PMC5977202; doi:10.3390/genes9050262)
Supplement: Supplementary file 1 [file genes-09-00262-s001.zip › DiapauseSelectionSIFigLegRev3.docx]

**Figure S1.** Map of the four paired sympatric collecting sites genotyped in the study, sampled from the Midwestern United States, where apple and hawthorn-infesting populations of *R. pomonella* co-occur. Also shown are the ranges of the apple (green) and hawthorn (red) host races in Canada and the United States. Note that the range of the apple host race is completely subsumed within the hawthorn host race range.

**Figure S2.** Relationships of the genetic responses of all 10,421 SNPs in the eclosion time GWAS (allele frequency difference between groups, early – late eclosing flies) versus geographic divergence (allele frequency difference between sites, Grant - Urbana) for: (a) the hawthorn race; (b) the apple race (as shown in [51]).
