# Supplementary material for: Genomic Differentiation during Speciation-with-Gene-Flow: Comparing Geographic and Host-Related Variation in Divergent Life History Adaptation in Rhagoletis pomonella
Source: Genes (Basel). 2018 May 18;9(5):262. doi: 10.3390/genes9050262 (PMC5977202; doi:10.3390/genes9050262)
Supplement: Supplementary file 1 [file genes-09-00262-s001.zip › DiapauseSelectionTableS4.docx]

**Table S4.** Correlation coefficients (r) of allele frequency responses in the eclosion time GWAS (Ecl.), apple prewinter selection experiment (A sel.), and hawthorn prewinter selection experiment (H sel.) with each other for All mapped SNPs (Map SNP) and for High, Intermediate (Int.), and Low LD classes of SNPs. Results are given for each chromosome considered separately, as well as all together (chr 1-5). ^*^ **=** P < 0.01; ^**^ = P < 0.01; ^***^ = P < 0.001; ^****^ = P < 0.0001; significant relationships positive in sign are highlighted in grey, while significant negative relationships have boxes boarded with dark lines. n = # of SNPs genotyped in the class.

|  | **chr 1** | **chr 2** | **chr 3** | **chr 4** | **chr 5** | **chr 1-5** |
| --- | --- | --- | --- | --- | --- | --- |
| Map SNP | n = 949 | n = 675 | n = 996 | n = 436 | n = 1188 | n = 4244 |
| Ecl. v A sel. | -0.28 | 0.68^****^ | 0.51^**^ | 0.01 | 0.06 | 0.26^*^ |
| Ecl. v H sel. | 0.07 | -0.35^*^ | 0.06 | -0.02 | 0.12 | -0.06 |
| H sel. v A sel. | -0.01 | -0.20 | 0.04 | -0.02 | -0.07 | -0.07 |
|  |  |  |  |  |  |  |
| High LD | n = 263 | n = 129 | n = 223 | n = 42 | n = 374 | n = 1031 |
| Ecl. v A sel. | 0.07 | 0.84^***^ | 0.08 | -0.08 | -0.07 | 0.40^*^ |
| Ecl. v H sel. | -0.09 | -0.40^*^ | -0.03 | 0.14 | -0.01 | -0.39^*^ |
| H sel. v A sel. | -0.05 | -0.39^*^ | -0.01 | -0.02 | 0.01 | -0.24 |
|  |  |  |  |  |  |  |
| Int. LD | n = 558 | n = 459 | n = 599 | n = 159 | n = 593 | n = 2368 |
| Ecl. v A sel. | -0.17 | 0.59^***^ | 0.43^**^ | -0.01 | 0.11 | 0.28^***^ |
| Ecl. v H sel. | 0.05 | -0.30^*^ | 0.09 | -0.03 | 0.10 | -0.05 |
| H sel. v A sel. | -0.03 | -0.11 | 0.04 | 0.05 | -0.04 | -0.04 |
|  |  |  |  |  |  |  |
| Low LD | n = 128 | n = 87 | n = 174 | n = 235 | n = 221 | n = 845 |
| Ecl. v A sel. | -0.17 | 0.19 | 0.17 | 0.04 | 0.11 | 0.06 |
| Ecl. v H sel. | 0.02 | 0.01 | 0.03 | -0.10 | -0.05 | -0.03 |
| H sel. v A sel. | 0.18 | 0.03 | 0.03 | -0.05 | -0.02 | 0.02 |
