# Supplementary material for: Genomic Differentiation during Speciation-with-Gene-Flow: Comparing Geographic and Host-Related Variation in Divergent Life History Adaptation in Rhagoletis pomonella
Source: Genes (Basel). 2018 May 18;9(5):262. doi: 10.3390/genes9050262 (PMC5977202; doi:10.3390/genes9050262)
Supplement: Supplementary file 1 [file genes-09-00262-s001.zip › DiapauseSelectionTableS3.docx]

**Table S3.** Percentages of SNPs displaying significant responses in the eclosion time GWAS (Ecl.), apple prewinter selection experiment (A sel.), and hawthorn prewinter selection experiment (H sel.) for All Mapped SNPs (Map SNP), and High, Intermediate (Int.), and Low LD classes. Results are given for each chromosome separately, as well as all together (chr 1-5). * = P < 0.01; ** = P < 0.01; *** = P < 0.001; **** = P < 0.0001; Grey boxes exceed null expectations. n = # SNPs scored in classes.

| **Ecl.** | **chr 1** | **chr 2** | **chr 3** | **chr 4** | **chr 5** | **chr 1-5** |
| --- | --- | --- | --- | --- | --- | --- |
| Map SNP | n = 949 | n = 675 | n = 996 | n = 436 | n = 1188 | n = 4244 |
|  | 63.7^****^ | 47.4^****^ | 45.2^****^ | 4.6 | 4.5 | 34.2^****^ |
| High LD | n = 263 | n = 129 | n = 223 | n = 42 | n = 374 | n = 1031 |
|  | 95.1^****^ | 68.2^****^ | 90.1^****^ | 4.8 | 0.5 | 52.7^****^ |
| Int. LD | n = 558 | n = 459 | n = 599 | n = 159 | n = 593 | n = 2368 |
|  | 59.3^****^ | 47.9^****^ | 37.1^****^ | 2.5 | 6.4 | 34.4^****^ |
| Low LD | n = 128 | n = 87 | n = 174 | n = 235 | n = 221 | n = 845 |
|  | 18.8^****^ | 13.8^*^ | 16.9^***^ | 5.9 | 6.3 | 10.9^**^ |
| **A sel.** | **chr 1** | **chr 2** | **chr 3** | **chr 4** | **chr 5** | **chr 1-5** |
| Map SNP | n = 949 | n = 675 | n = 996 | n = 436 | n = 1188 | n = 4244 |
|  | 3.5 | 28.9^**^ | 8.9 | 3.2 | 2.3 | 8.4 |
| High LD | n = 263 | n = 129 | n = 223 | n = 42 | n = 374 | n = 1031 |
|  | 1.1 | 58.1^**^ | 9.4 | 0.0 | 0.0 | 9.6 |
| Int. LD | n = 558 | n = 459 | n = 599 | n = 159 | n = 593 | n = 2368 |
|  | 3.9 | 25.3^**^ | 8.8 | 1.3 | 3.0 | 8.9 |
| Low LD | n = 128 | n = 87 | n = 174 | n = 235 | n = 221 | n = 845 |
|  | 6.3 | 4.6 | 8.6 | 5.1 | 4.1 | 5.7 |
| **H sel.** | **chr 1** | **chr 2** | **chr 3** | **chr 4** | **chr 5** | **chr1-5** |
| Map SNP | n = 949 | n = 675 | n = 996 | n = 436 | n = 1188 | n = 4244 |
|  | 3.9 | 7.6 | 4.5 | 8.5 | 5.2 | 5.1 |
| High LD | n = 263 | n = 129 | n = 223 | n = 42 | n = 374 | n = 1031 |
|  | 0.0 | 4.7 | 0.0 | 0.0 | 1.6 | 1.2 |
| Int. LD | n = 558 | n = 459 | n = 599 | n = 159 | n = 593 | n = 2368 |
|  | 4.8 | 7.6 | 4.8 | 9.4 | 7.3 | 6.3 |
| Low LD | n = 128 | n = 87 | n = 174 | n = 235 | n = 221 | n = 845 |
|  | 7.8 | 11.5 | 9.2 | 9.4 | 5.9 | 8.4 |
