# Supplementary material for: Genomic Differentiation during Speciation-with-Gene-Flow: Comparing Geographic and Host-Related Variation in Divergent Life History Adaptation in Rhagoletis pomonella
Source: Genes (Basel). 2018 May 18;9(5):262. doi: 10.3390/genes9050262 (PMC5977202; doi:10.3390/genes9050262)
Supplement: Supplementary file 1 [file genes-09-00262-s001.zip › DiapauseSelectionTableS2.docx]

**Table S2.** Synopsis of prior work and sources of additional data sets analyzed in the current study.

| **Citation** | **New data** | **Additional data used** | **Summary** |
| --- | --- | --- | --- |
| Egan et al. 2015 | - Hawthorn selection experiment - Grant, MI host races | --- | Hawthorn flies surviving longer prewinter periods displayed significant genome-wide shifts in SNP frequencies toward the apple race (chr. 1-5, in all LD classes). |
| Ragland et al. 2017 | - Eclosion GWAS - Fennville, MI host races | - Hawthorn selection experiment | Variation underlying eclosion timing is concentrated on chr. 1-3, particularly in the high LD regions, putatively associated with inversions. Eclosion variation is largely genomically independent from diapause intensity in hawthorn race. |
| Doellman et al. under review | - Grant, MI host races Fennville, MI host races - Dowagiac, MI host races - Urbana, MI host races | - Eclosion GWAS | Geographic variation within host races is highly correlated with eclosion variation, particularly on chr. 1-3 in the hawthorn race and chr. 2-3 in the apple race. |
| Current study | - Apple selection experiment | - Hawthorn selection experiment - Eclosion GWAS - Grant, MI host races - Fennville, MI host races - Dowagiac, MI host races - Urbana, MI host races | SNPs in the high and intermediate LD regions on chr. 2 responded significantly in the apple selection experiment. The majority of the genome showing significant geographic and host-related variation can be accounted for by loci putatively under selection for initial diapause intensity and eclosion time phenotypes. |
