# Supplementary material for: Genomic Differentiation during Speciation-with-Gene-Flow: Comparing Geographic and Host-Related Variation in Divergent Life History Adaptation in Rhagoletis pomonella
Source: Genes (Basel). 2018 May 18;9(5):262. doi: 10.3390/genes9050262 (PMC5977202; doi:10.3390/genes9050262)
Supplement: Supplementary file 1 [file genes-09-00262-s001.zip › DiapauseSelectionTableS1.docx]

**Table S1.** Host plant origin, numerical designation of sites, location, latitude [N] and longitude [W] in degrees, year sampled, and number of individuals genotyped (*n*) for the four pairs of sympatric hawthorn and apple fly populations analyzed in the study across the Midwestern USA. See Figure S1 for a map of the sites.

| **Host** | | **Site #** | | **Location** | **Lat., Long.** | **Year** | | ***n*** |  |  |
| --- | --- | --- | --- | --- | --- | --- | --- | --- | --- | --- |
|  | | Haw Hawthorn (*Crataegus mollis*) | | 1 | Grant, Newaygo Co., MI | 43.35, 85.9 | | 1989 | 54 | |
|  | |  | | 2 | Fennville, Allegan Co., MI | 42.6, 86.15 | | 2008 | 96 | |
|  | |  | | 3 | Dowagiac, Cass Co., MI | 41.88, 86.23 | | 2006 | 32 | |
|  | |  | | 4 | Urbana, Champaign Co., IL | 40.08, 88.23 | | 2000 | 48 | |
|  | | Apple (*Malus domestica*) | | 1 | Grant, Newaygo Co., MI | 43.35, 85.9 | | 1989 | 48 | |
|  | |  | | 2 | Fennville, Allegan Co., MI | 42.6, 86.15 | | 2008 | 93 | |
|  | |  | | 3 | Dowagiac, Cass Co., MI | 41.88, 86.23 | | 2006 | 32 | |
|  | |  | | 4 | Urbana, Champaign Co., IL | 40.08, 88.23 | | 2000 | 38 | |
