# Supplementary material for: Genomic Differentiation during Speciation-with-Gene-Flow: Comparing Geographic and Host-Related Variation in Divergent Life History Adaptation in Rhagoletis pomonella
Source: Genes (Basel). 2018 May 18;9(5):262. doi: 10.3390/genes9050262 (PMC5977202; doi:10.3390/genes9050262)
Supplement: Supplementary file 1 [file genes-09-00262-s001.zip › DiapauseSelectionTableS7.docx]

**Table S7.** Correlation coefficients (r) of SNP allele frequency differences between 7-day and 32-day treatments in the hawthorn prewinter selection experiment (H sel. exp.) versus geographic differences between apple race (upper table) and hawthorn race (lower table) populations from Grant, MI and Urbana, IL. for All Mapped SNPs (Map SNP), and for High, Intermediate (Int.), and Low LD classes of SNPs. Results are given for each chromosome considered separately, as well as for all together (chr 1-5). ^*^ = P < 0.05; ^**^ = P < 0.05; ^**^ = P < 0.01; ^***^ = P < 0.001; ^****^ = P < 0.0001; significant positive relationships are shaded in grey. n = # of SNPs genotyped in classes.

| **Apple race** | **chr 1** | **chr 2** | **chr 3** | **chr 4** | **chr 5** | **chr 1-5** |
| --- | --- | --- | --- | --- | --- | --- |
| Map SNPs | n = 949 | n = 675 | n = 996 | n = 436 | n = 1188 | n = 4244 |
|  | 0.01 | -0.25 | 0.06 | 0.09 | 0.11 | -0.01 |
| High LD | n = 263 | n = 129 | n = 223 | n = 42 | n = 374 | n = 1031 |
|  | 0.03 | -0.28 | 0.08 | -0.05 | -0.07 | -0.20 |
| Med LD | n = 558 | n = 459 | n = 599 | n = 159 | n = 593 | n = 2368 |
|  | 0.02 | -0.19 | 0.05 | 0.06 | 0.10 | 0.01 |
| Low LD | n = 128 | n = 87 | n = 174 | n = 235 | n = 221 | n = 845 |
|  | -0.05 | -0.08 | 0.07 | -0.12 | 0.15 | 0.01 |
| **Haw race** | **chr 1** | **chr 2** | **chr 3** | **chr 4** | **chr 5** | **chr 1-5** |
| Map SNPs | n = 949 | n = 675 | n = 996 | n = 436 | n = 1188 | n = 4244 |
|  | 0.17 | -0.09 | 0.28 | 0.30 | 0.35 | 0.16^*^ |
| High LD | n = 263 | n = 129 | n = 223 | n = 42 | n = 374 | n = 1031 |
|  | 0.11 | -0.21 | 0.27^*^ | 0.33 | 0.25 | -0.23 |
| Med LD | n = 558 | n = 459 | n = 599 | n = 159 | n = 593 | n = 2368 |
|  | 0.17 | -0.18 | 0.30^*^ | 0.35 | 0.24 | 0.17 |
| Low LD | n = 128 | n = 87 | n = 174 | n = 235 | n = 221 | n = 845 |
|  | 0.24 | 0.21 | 0.48^****^ | 0.27^**^ | 0.43^****^ | 0.34^****^ |
