# Supplementary figures and images for: Genomic Differentiation during Speciation-with-Gene-Flow: Comparing Geographic and Host-Related Variation in Divergent Life History Adaptation in Rhagoletis pomonella
Source: Genes (Basel). 2018 May 18;9(5):262. doi: 10.3390/genes9050262 (PMC5977202; doi:10.3390/genes9050262)

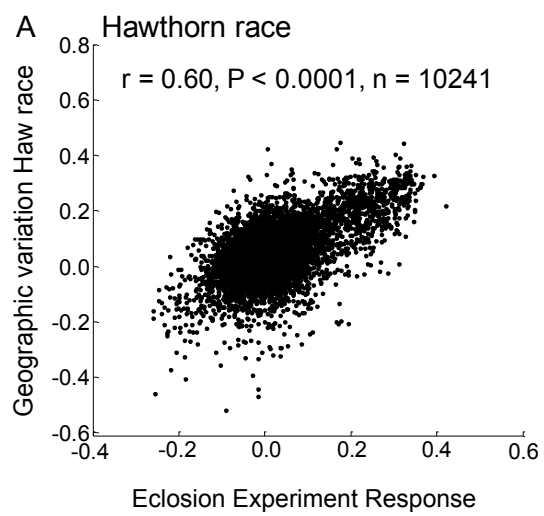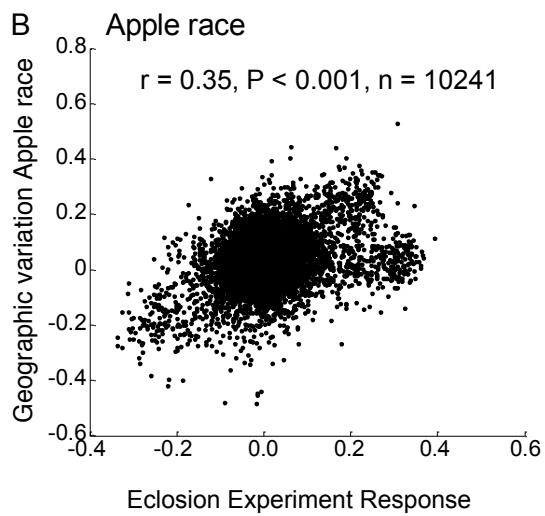

Supplement: Supplementary file 1 [file genes-09-00262-s001.zip › DiapauseSelectionFigS2rev3.pdf]

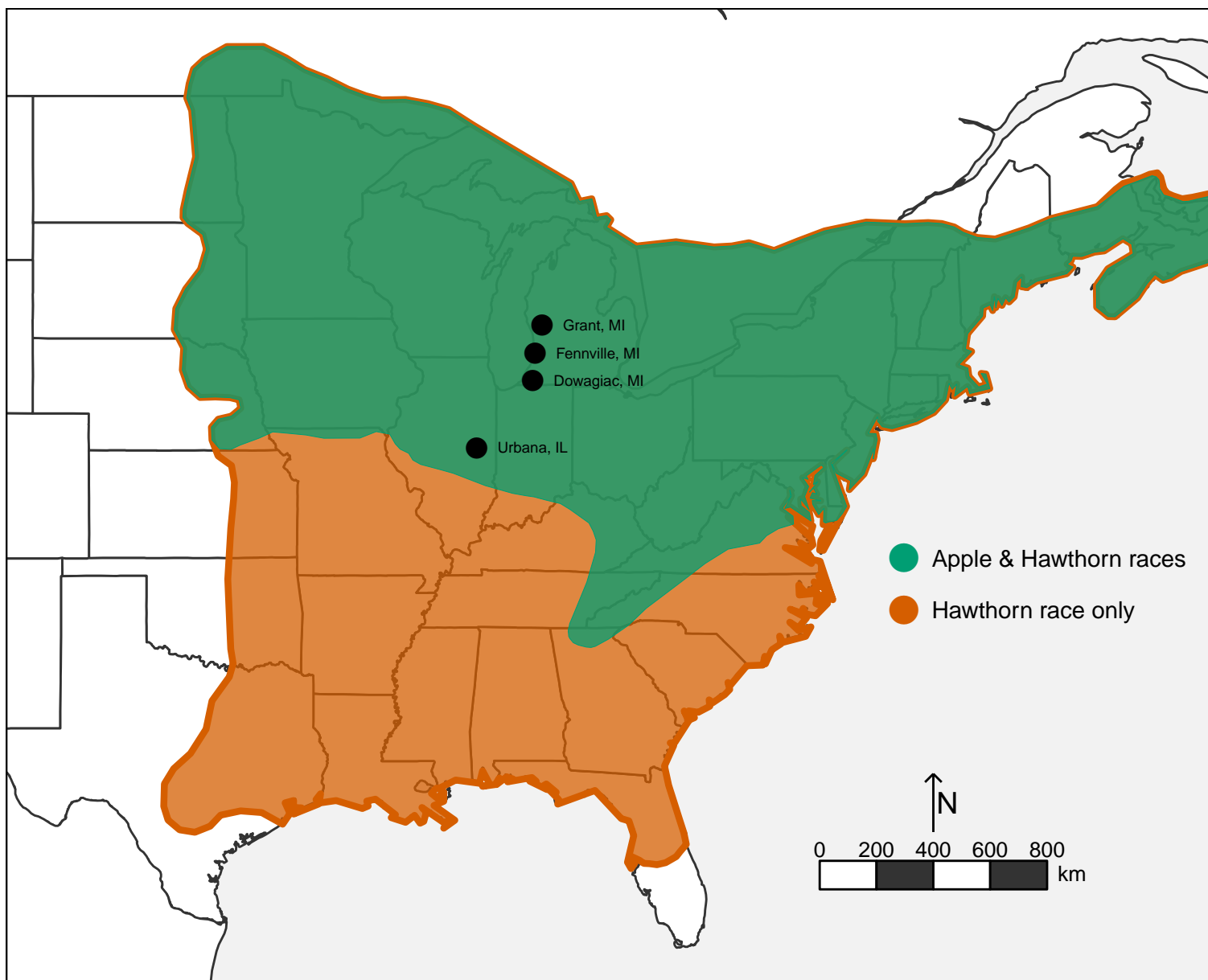

Supplement: Supplementary file 1 [file genes-09-00262-s001.zip › DiapauseSelectionFigS1rev3.pdf]
